# Inulin ameliorates metabolic syndrome in high-fat diet-fed mice by regulating gut microbiota and bile acid excretion

**DOI:** 10.3389/fphar.2023.1226448

**Published:** 2023-07-24

**Authors:** Shaoxiong Huang, Shiliang Dong, Lizhen Lin, Qiming Ma, Mengping Xu, Limei Ni, Qitong Fan

**Affiliations:** ^1^ Department of Gastrointestinal Surgery, Affiliated Hospital of Putian University, Putian, China; ^2^ Department of Bariatric Surgery, The First Affiliated Hospital of Jinan University, Guangzhou, China; ^3^ Department of Anaesthesia, The First Hospital of Putian City, Putian, Fujian, China; ^4^ The Department of General Surgery, The First Affiliated Hospital of Gannan Medical University, Ganzhou, Jiangxi, China; ^5^ Department of General Practice, Affiliated Hospital of Putian University, Putian, China; ^6^ Department of Human Anatomy, Nanchang University Fuzhou Medical College, Fuzhou, China

**Keywords:** Intestine, gut microbes, bile acids, inulin, high-fat diet

## Abstract

**Background:** Inulin is a natural plant extract that improves metabolic syndrome by modulating the gut microbiota. Changes in the gut microbiota may affect intestinal bile acids. We suggest that inulin may improve metabolism by inducing bile acid excretion by gut microbes.

**Methods:** Male C57/BL mice were fed either a high-fat diet (60% calories) or a regular diet for 16 weeks, with oral inulin (10% w/w). At the end of the experiment, the gene expression levels (FGF15, CD36, Srebp-1c, FASN, and ACC) in the liver and intestines, as well as the serum levels of triglycerides (TGs), low-density lipoprotein (LDL) cholesterol, total cholesterol, and free fatty acids, were collected. The expression of FGF15 was examined using Western blot analysis. The fat distribution in the liver and groin was detected by oil red and hematoxylin and eosin staining. Simultaneously, the levels of serum inflammatory factors (alanine aminotransferase and aspartate aminotransferase) were detected to explore the side effects of inulin.

**Results:** Inulin significantly improved glucose tolerance and insulin sensitivity, and decreased body weight and serum TG and LDL levels, in mice fed normal diet. Furthermore, inulin increased the α-diversity of the gut microbiota and increased the fecal bile acid and TG excretion in inulin-treated mice. In addition, inulin significantly reduced lipid accumulation in liver and inguinal fat, white fat weight, and hepatic steatosis. Western blot analysis showed that inulin reduced the expression of FGF15, a bile acid reabsorption protein.

**Conclusion:** Inulin ameliorates the glucose and lipid metabolic phenotypes of mice fed a normal diet, including decreased intestinal lipid absorption, increased glucose tolerance, increased insulin sensitivity, and decreased body weight. These changes may be caused by an increase in bile acid excretion resulting from changes in the gut microbiota that affect intestinal lipid absorption.

## 1 Introduction

Nonalcoholic fatty liver disease (NAFLD), including alcoholic fatty liver disease and nonalcoholic steatohepatitis, is a risk factor for hepatocellular carcinoma and is characterized by excessive triglyceride (TG) accumulation within hepatocytes (Brunt et al., 2015; Lambertz et al., 2017). The prevalence of obesity leads to an increased global prevalence of liver cancer, in which obese patients are more likely to develop fatty liver and liver damage (Porras et al., 2018; Younossi et al., 2019). There are many factors that influence the occurrence and development of obesity, including genetic, environmental, and commensal microbial factors (Murea et al., 2012). Increasing evidence suggests that the gut microbiota plays a critical role in the control of obesity and NAFLD (Duranti et al., 2017; Soderborg et al., 2018). Microbial derivatives, including short-chain fatty acids, lipopolysaccharides, and secondary bile acids, are involved in the regulation of hepatic lipid metabolism.

Inulin is a natural plant extract and a fermentable linear polysaccharide composed of fructan and glucose. It is a prebiotic and has been reported to reduce weight gain, liver weight, and plasma and liver TG levels to decrease hepatic steatosis in a mouse model of high-fat-induced obesity (van der Beek et al., 2018; Beisner et al., 2021). Previous studies have shown that prebiotics, such as oligosaccharides and inulin, change the concentration of microbial derivatives by modulating the microbial community structure, thereby controlling body weight and reducing hepatic fat accumulation (Hu et al., 2023a). Recent clinical data also confirm that inulin improves metabolic syndrome by regulating changes in the gut microbiota (Nicolucci et al., 2017). The function of bile acids in the gut is to promote fat absorption, and reabsorption favors the intestinal-hepatic circulation (Mori et al., 2022). A growing body of research confirms that the gut microbiota regulates intestinal lipid absorption by affecting the gut-hepatic circulation of bile acids (Mori et al., 2022).

Inulin, a prebiotic, does not influence the intestinal and hepatic circulation of bile acids. Our aim was to investigate the macro-effects of inulin on gut microbes and the function of bile acid reabsorption in the gut. In addition, the effects of a high-fat diet (HFD) on the metabolic phenotype of mice were studied.

## 2 Materials and methods

### 2.1 Animal experiments

C57BL mice (17 ± 1.5 g) (*n* = 40) were obtained from the Experimental Animal Center, Fuzhou Medical College, Nanchang University, Fuzhou, Jiangxi, China. The animals were maintained at a controlled temperature (22°C ± 1°C), humidity (50%), and light (12/12-h light/dark). All animal experiments were approved by the Experimental Animal Ethics Committee of Fuzhou Medical College, Nanchang University. Inulin was obtained from MCE (HY-N7075) and dissolved in drinking water (10% w/w). The blood glucose testing equipment was offered by Sinocare. The TG, low-density lipoprotein (LDL), high-density lipoprotein, cholesterol, free fatty acids, interleukin-6, interleukin-1β, and tumor necrosis factor testing kits were obtained from Nanjing Jiancheng Company. The insulin ELISA kit was purchased from Abcam. The HFD consisted of 20% carbohydrates, 35% proteins, and 45% fats (D12492; Research Diets; 60% of total calories).

### 2.2 Analysis of serum and fecal lipids

After the mice were fasted for 6 h, blood samples were obtained from the orbit of the mice. Then, 5,000 g of the supernatant was centrifuged and stored at −80° until use. According to the manufacturer’s recommendations, the TG, LDL, high-density lipoprotein, cholesterol, free fatty acids, interleukin-6, interleukin-1β, and tumor necrosis factor levels were evaluated. For the fecal lipid test, the mice were placed separately and samples were collected for a 24-h fecal test. Then, the 24-h triglyceride level was calculated, according to the manufacturer’s instructions.

### 2.3 Real-time polymerase chain reaction (PCR)

Fresh tissues were ground and crushed, and total RNA was prepared using the TRIzol reagent (Ambion). The amplified products were labeled with SYBR (Yeasen, 10222ES60). RT-PCR was performed using the Bio-rad PCR system. The relative mRNA levels were calculated by comparison using the threshold cycle method. The primer sequences are presented in Table 1 **.**

**TABLE 1:** Primer information table.

Gene	Forword	Reverse
Chrebp	CCA​GCC​TCA​AGG​TGA​GCA​AA	CAT​GTC​CCG​CAT​CTG​GTC​A
Srebp1c	GCA​GCC​ACC​ATC​TAG​CCT​G	CAG​CAG​TGA​GTC​TGC​CTT​GAT
Apob48	TTG​GCA​AAC​TGC​ATA​GCA​TCC	TCA​AAT​TGG​GAC​TCT​CCT​TTA​GC
Fasn	AGA​GAT​CCC​GAG​ACG​CTT​CT	GCT​TGG​TCC​TTT​GAA​GTC​GAA​GA
Acc	CTC​CCG​ATT​CAT​AAT​TGG​GTC​TG	TCG​ACC​TTG​TTT​TAC​TAG​GTG​C
Scd-1	TTC​TTG​CGA​TAC​ACT​CTG​GTG​C	CGG​GAT​TGA​ATG​TTC​TTG​TCG​T
Cd36	ATG​GGC​TGT​GAT​CGG​AAC​TG	TTT​GCC​ACG​TCA​TCT​GGG​TTT
Npc1l1	TGT​CCC​CGC​CTA​TAC​AAT​GG	CCT​TGG​TGA​TAG​ACA​GGC​TAC​TG
Tnf-α	CAG​GCG​GTG​CCT​ATG​TCT​C	CGA​TCA​CCC​CGA​AGT​TCA​GTA​G
Il-6	TAG​TCC​TTC​CTA​CCC​CAA​TTT​CC	TTG​GTC​CTT​AGC​CAC​TCC​TTC
Il-1β	GAA​ATG​CCA​CCT​TTT​GAC​AGT​G	TGG​ATG​CTC​TCA​TCA​GGA​CAG
Fgf15	GGT​CCC​TAT​GTC​TCC​AAC​TGC	CTT​GAT​GGC​AAT​CGT​CTT​CAG​A

### 2.4 Glucose tolerance and insulin tolerance tests

Before the glucose tolerance test, the mice were fasted for 16 h and injected with 2 g/kg body weight of glucose. Blood glucose levels in the rat tail blood were measured at 0, 15, 30, 60, and 120 min. Mice were fasted for 6 h before the insulin resistance test. Blood glucose levels were measured at 0, 15, 30, 60, and 120 min after injection of 0.55 µ/kg body weight.

### 2.5 Histological staining

Fresh liver and white adipose tissues were fixed with 4% paraformaldehyde for 24 h and embedded with Oct. Then, the samples were sectioned at a thickness of 4 µm using a Thermo Fisher Scientific Slicer and stored at −80°. For hematoxylin and eosin staining, the cut tissue sections were washed three times with phosphate-buffered saline (PBS), placed into the hematoxylin solution for 1 min, washed three times with PBS, placed into the eosin solution for 1 min, washed three times with PBS, and sealed with xylene transparent and neutral gum. The samples were photographed under a Leica microscope. For oil-red staining, the cut tissue sections were washed three times with PBS, stained with oil red solution for 1 min, washed three times with PBS, washed three times with hematoxylin solution for 1 min, washed three times with PBS, and sealed with glycerol gelatin. The sections were photographed under a Leica microscope. ImageJ software was used to calculate the oil red-stained area.

### 2.6 Western blot analysis

RIPA lysis buffer was used to extract intestinal epithelial tissue protein. BCA protein assay kit was used to determine the protein concentrations (Beyotime, Shanghai, China). The membrane was sealed with 5% BSA for 2 h. The membrane was subsequently compared with the target Fgf15 (1:1000, ab252922) at 4°C. GAPDH (1:1000) was used as a loading control. Then, the secondary antibody (1:2000) was allowed to bind to the primary antibody for 2 h at room temperature. The signal was captured using an emitter-coupled logic substrate (pierce chemical). ImageJ software was used to quantitatively analyze the band intensity.

### 2.7 Detection of total bile acids

Serum and fecal total bile acid levels were determined using total bile acid kits (e003-2-1; Nanjing Jiancheng Co., Ltd.).

### 2.8 Statistical analysis

Data are expressed as the mean ± standard error of the mean (SEM). Furthermore, significant differences were determined by performing a *t*-test with least significant difference (LSD) *post hoc* tests, and statistical significance was set at *p* < 0.05.

## 3 Results

### 3.1 Inulin improved glucose and lipid metabolism in mice fed normal diet

**FIGURE 1:**
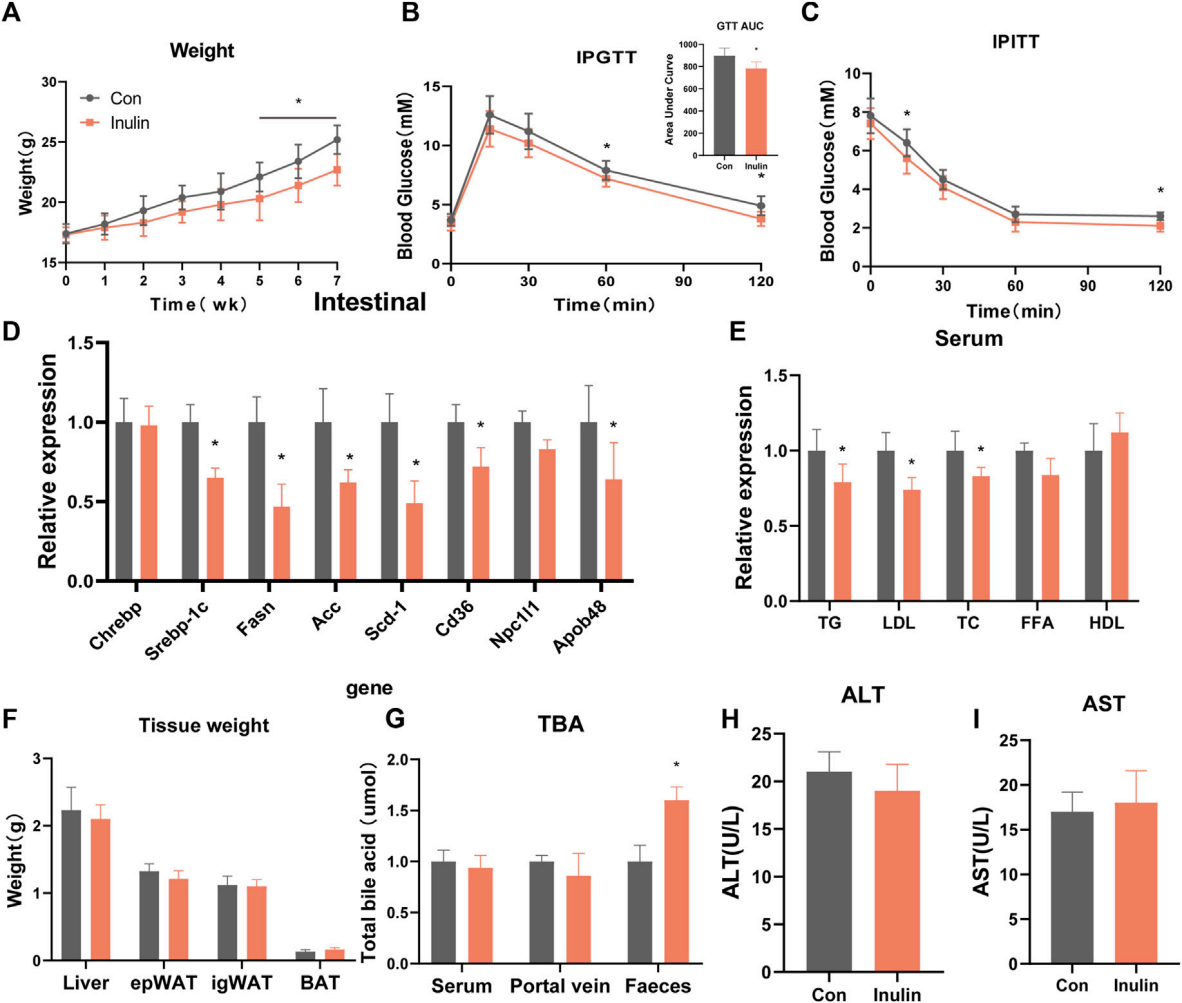
Phenotype of mice fed a normal diet with inulin. **(A)** Body weight of mice on normal diet, **(B)** glucose tolerance test, **(C)** insulin tolerance test, **(D)** jejunal gene expression, **(E)** serum lipid levels, **(F)** tissue weight, **(G)** total bile acid content, **(H)** alanine transaminase, **(I)** aspartate transaminase.

To understand the effects of inulin on glucose and lipid metabolism, we fed inulin to mice on a regular diet for 7 weeks, while the control group was fed with the same quantity of solvent. The results showed that the body weight of inulin-fed mice was significantly lower than that of the control group (*p* < 0.05; Figure 1A). Inulin significantly improved glucose tolerance and insulin sensitivity in mice fed a normal diet. The area of the glucose tolerance test curve also confirmed that the inulin group significantly improved the glucose tolerance (*p* < 0.05; Figures 1B, C). To determine the effect of inulin on intestinal lipid synthesis and transport, we examined the genes involved in inulin-fed small intestinal epithelial cells in mice. The results showed that the expressions of some lipid synthesis genes (Srebp-1c, Fasn, Acc, and Scd-1) and lipid transport genes (Cd36 and Apob-48) were significantly lower in inulin-fed mice than those in control mice (Figure 1D). To observe the serum lipid content in inulin-fed mice, we measured the serum levels of TG, LDL, cholesterol, free fatty acids, and high-density lipoprotein. The results showed that the serum levels of TG, LDL, and cholesterol were significantly lower in inulin-fed mice than those in control mice (*p* < 0.05; Figure 1E). In addition, we did not find any significant changes in the liver, inguinal fat (igWAT), epididymal fat (Epwat), or brown fat (BAT) when the mice were weighed (Figure 1F). To understand the effect of inulin feeding on intestinal bile acid excretion, we examined the total bile acid levels in the serum, portal vein, and feces of mice. The results showed that inulin significantly promoted fecal bile acid excretion (*p* < 0.05; Figure 1G). To observe the effect of inulin on liver function, serum alanine aminotransferase and aspartate aminotransferase levels were detected; however, no significant differences were observed (Figures 1H, I).

### 3.2 Inulin enhances microbial diversity in the gut

**FIGURE 2:**
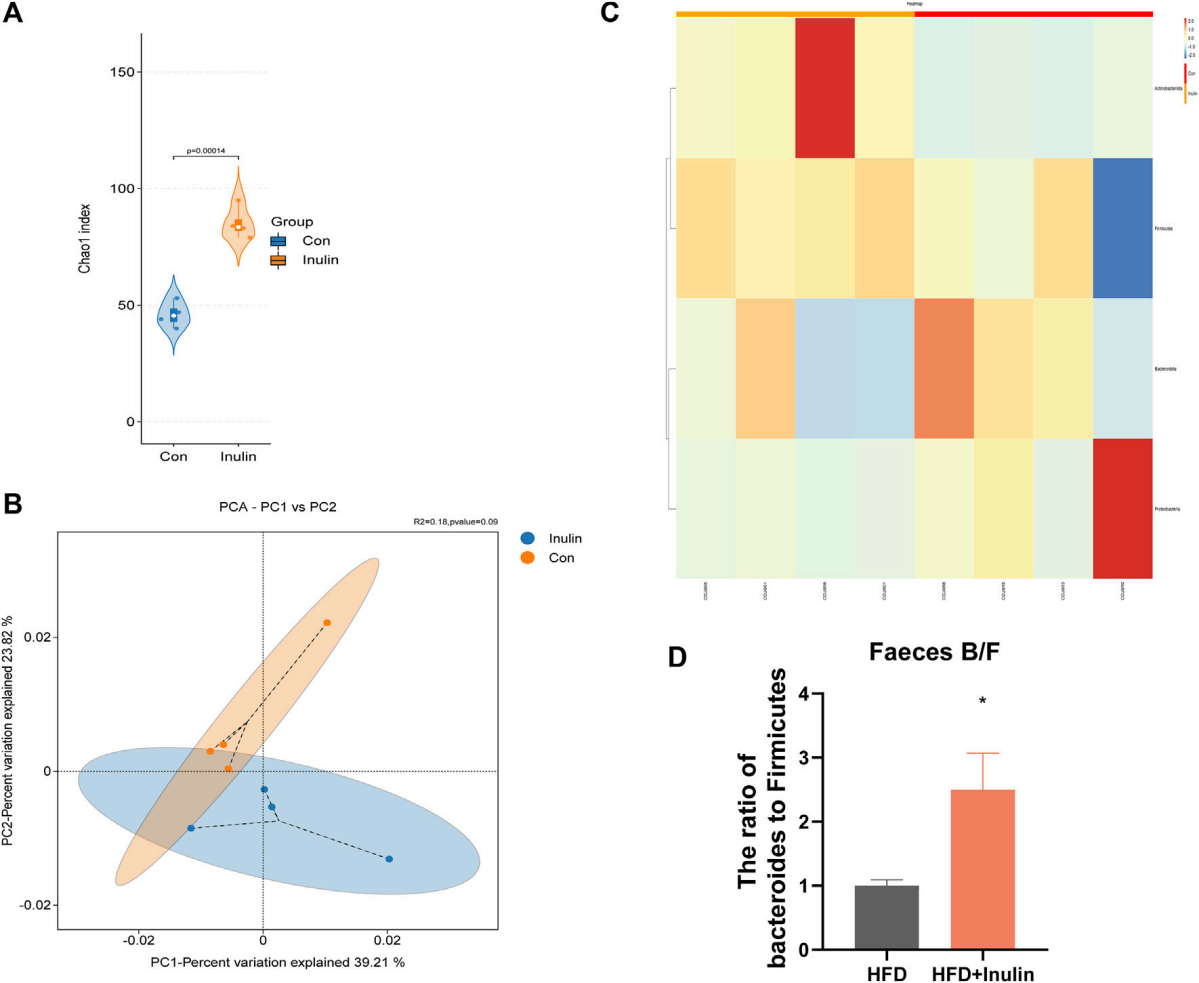
Sequencing results of gut microbes from mice fed a normal diet. **(A)** Microbial α-diversity; **(B)** microbial β-diversity; **(C)** changes in levels of microbial “Phyla”; **(D)** proportion of *bacteroides* and *firmicutes.*

To further understand the effect of inulin on the gut microbiota, we performed 16sRNA sequencing of intestinal contents in mice fed a normal diet. We found that inulin increased the α-diversity of the gut microbiota in mice (Figure 2A). At the same time, the principal component analysis showed that inulin increased the β-diversity of the gut microbes in mice (Figure 2B). To further evaluate the microbial changes, we analyzed the microbial “Phylum” levels and found that inulin increased the proportion of the Bacteroidetes enteritidis and decreased the proportion of Firmicutes. Furthermore, the ratio of *bacteroides* to Firmicutes was significantly increased (Figures 2C, D).

### 3.3 Inulin reduced intestinal bile acid reabsorption and increased fecal TG excretion

**FIGURE 3:**
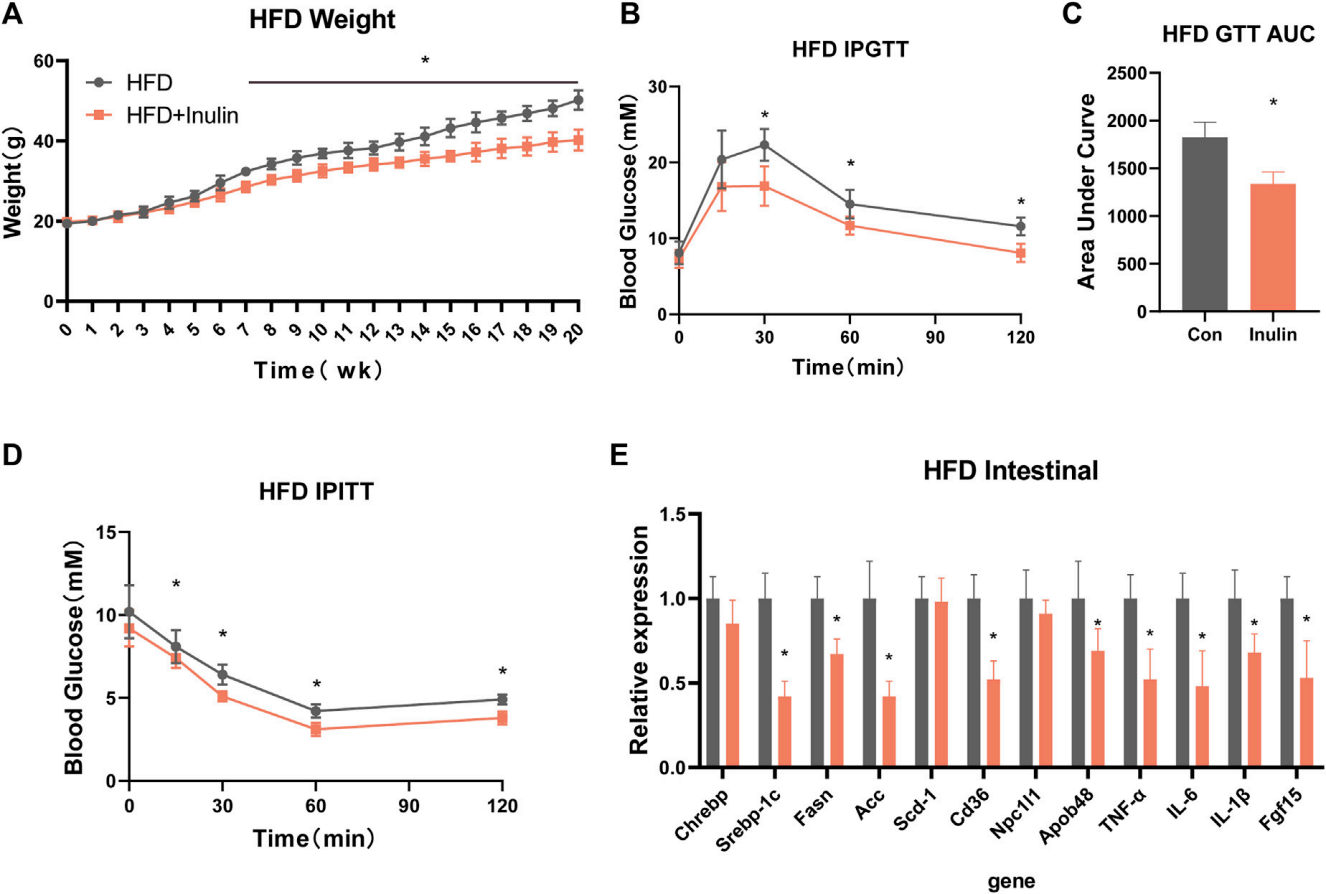
Metabolic phenotype after inulin-fed mice were fed a high-fat diet. **(A)** Body weight of mice on a high-fat diet; **(B)** glucose tolerance test; **(C)** area under the curve of glucose tolerance test; **(D)** insulin tolerance test; **(E)** intestinal lipid absorption-related genes in mice on a high-fat diet.

**FIGURE 4:**
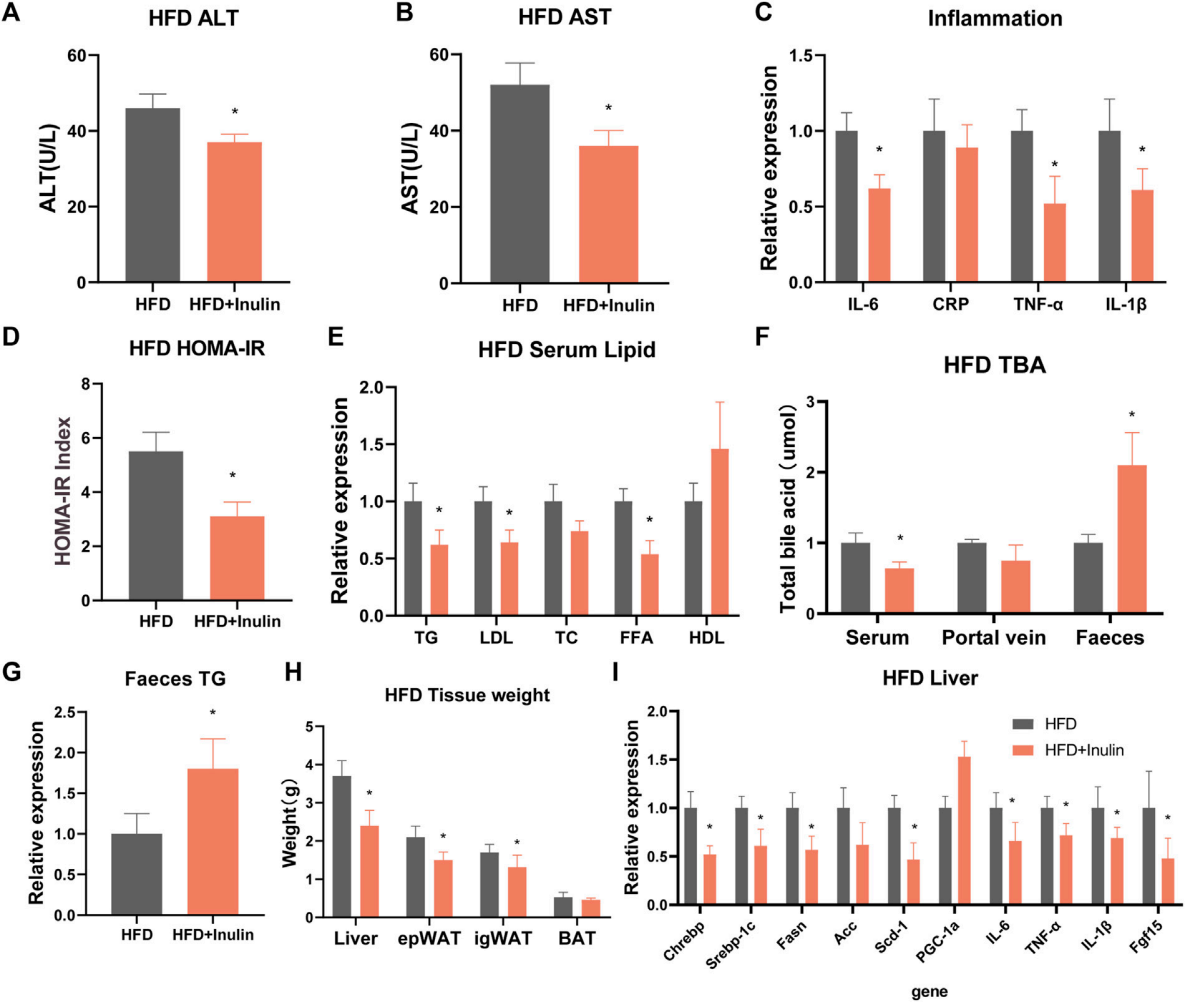
Serological detection and liver gene expression in mice fed a high-fat diet. **(A)** Alanine transaminase; **(B)** aspartate transaminase; **(C)** serum inflammatory factors; **(D)** insulin homeostasis model (HOMA-IR); **(E)** serum lipid levels; **(F)** total bile acid levels; **(G)** fecal triglyceride content; **(H)** tissue weight of mice on a high-fat diet; and **(I)** expression of liver-related genes.

To further explore the effects of inulin on glycolipid metabolism, we used mice fed a HFD and inulin or solvent as experimental treatments. The results showed that inulin significantly reduced the weight in mice fed a HFD, which was significantly lower at 7 weeks than the control group (Figure 3A). Glucose tolerance and insulin resistance tests were performed 20 weeks after feeding a HFD. The results showed that inulin significantly improved the glucose tolerance and insulin resistance symptoms associated with a HFD (Figures 3B–D). To further evaluate the effects of inulin on intestinal absorption in mice fed a HFD, we examined the lipid and bile acid reabsorption genes in small intestinal epithelial cells. The results showed that inulin significantly decreased intestinal lipid absorption (Srebp-1c, Fasn, ACC, CD36, NPC1L1, and APOB48) and the expression of inflammatory genes (TNF-α, IL-6, and IL-1β) (*p* < 0.05). In addition, inulin reduced the expression of the bile acid reabsorption gene (FGF15) (*p* < 0.05; Figure 3E). To determine the effect of inulin on serum lipids and inflammation in mice fed a HFD, we measured serum levels of lipids, total bile acids, and inflammatory markers. The results showed that inulin decreased the serum levels of alanine aminotransferase, aspartate aminotransferase, IL-6, IL-1β, and TNF-α (*p* < 0.05) in mice fed with HFD (Figures 4A–C). Furthermore, inulin improved the insulin resistance index (*p* < 0.05) in mice fed a HFD (Figure 4D). The levels of TG, LDL, and FFA were significantly lower in the inulin group than those in the control group (*p* < 0.05) (Figure 4E). Interestingly, inulin-fed mice on a HFD had lower levels of serum total bile acids, whereas fecal excretion of bile acids was higher (*p* < 0.05) (Figure 4F). To this end, we examined the fecal TG content and found that the fecal TG content was significantly higher in inulin-fed mice fed a HFD (*p* < 0.05) (Figure 4G). We further weighed the liver and fat of mice on a HFD and found that inulin reduced the weight of liver, inguinal fat (igWAT), and epididymal fat (EPWAT) (*p* < 0.05) (Figure 4H). To further explore the beneficial effect of inulin on the liver, we examined liver-associated genes for lipid absorption and inflammation. Compared with the control group, inulin reduced the steatosis lipid synthesis genes (CHREBP, Srebp-1c, FASN, and Scd-1) in mice fed a HFD and also significantly decreased the expressions of IL-6, IL-1β, and TNF-1α in the liver. In addition, inulin reduced the expression of FGF15, a rate-limiting gene for bile acid synthesis in the liver (*p* < 0.05; Figure 4I). These results suggest that inulin improves the glycolipid metabolic phenotype of mice fed a HFD by reducing the reabsorption of intestinal bile acids and TG excretion from the feces, reducing the metabolic syndrome induced by a HFD.

### 3.4 Inulin reduced liver lipid accumulation and white fat production

**FIGURE 5:**
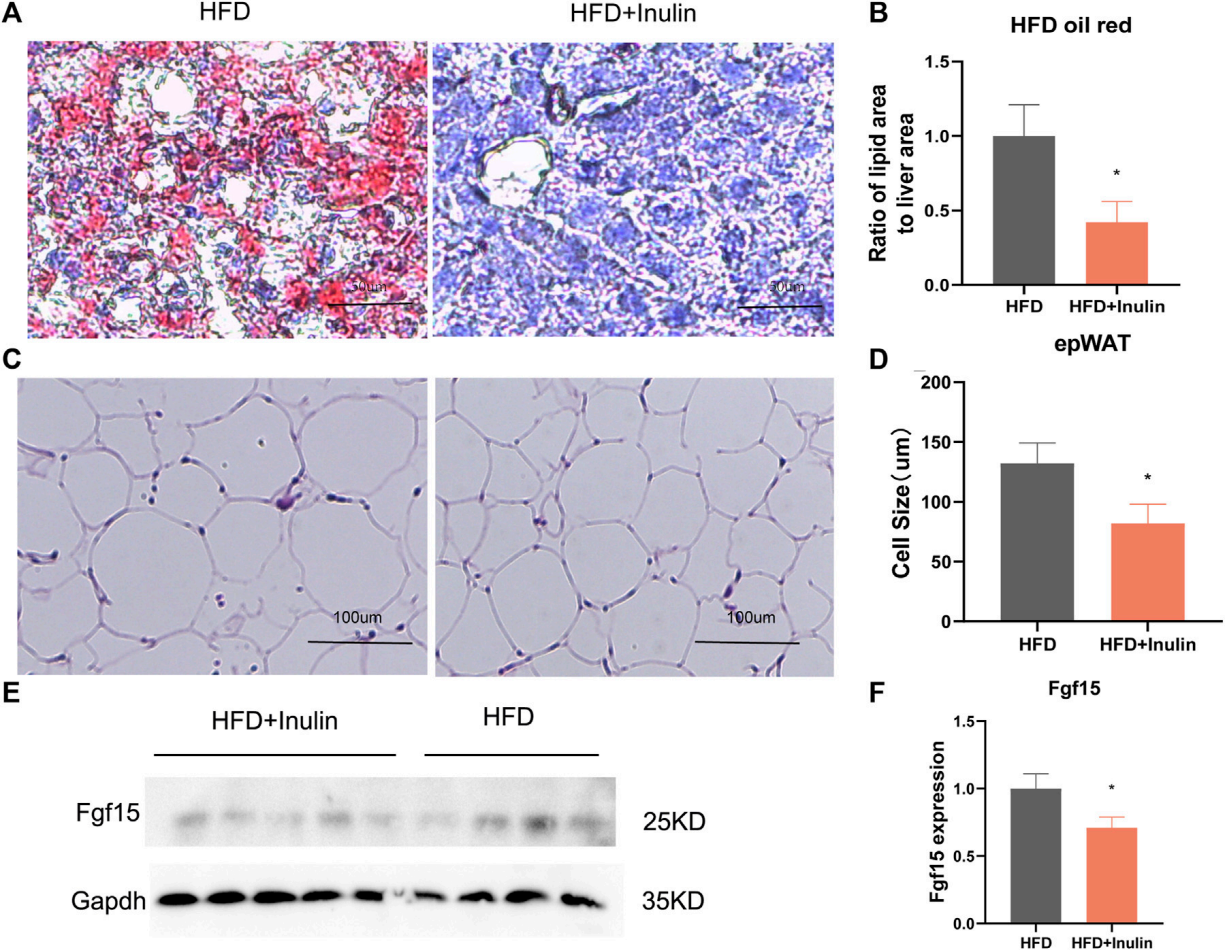
Tissue sections and protein expression of mice on a high-fat diet. **(A)** Liver oil-red staining; **(B)** lipid proportion statistics after liver oil-red staining; **(C)** groin fat hematoxylin and eosin staining; **(D)** groin fat cell size statistics; **(E)** Western blot plot of FGF15, the intestinal bile acid reabsorption protein; **(F)** protein expression statistics of intestinal bile acid reabsorption protein FGF15.

To further explore the benefit of inulin on lipid accumulation in the liver, we performed oil-red staining of liver samples of inulin- and HFD-fed mice and analyzed the liver lipid proportion. The results showed that inulin significantly reduced the liver lipid accumulation (*p* < 0.05; Figures 5A, B) in mice fed a HFD. To observe the maturity of white fat, we performed hematoxylin and eosin staining of inguinal fat and found that inulin significantly reduced the diameter of inguinal fat in mice fed a HFD. The inguinal fat size of inulin-fed mice on a HFD was 40% smaller than that of controls (*p* < 0.05; Figures 5C, D). To further determine the mechanism of bile acid reabsorption in the gut, we examined the expression level of FGF15, a gut bile acid reabsorption protein. We found that inulin significantly reduced the expression of intestinal bile acid reabsorption protein FFG15 in mice fed a high-fat diet. These results suggest that the inhibitory effect of inulin on the intestinal bile acid reabsorption protein FGF15 leads to a reduction in intestinal lipid absorption, which in turn affects lipid production and accumulation of liver and white fat (*p* < 0.05; Figures 5E, F).

## 4 Discussion

The present results show that inulin improves glycolipid metabolism by remodeling the gut microbiota and increasing fecal bile acid excretion in mice. In particular, inulin ameliorated the glycolipid metabolic phenotype of mice fed a normal diet, which included reduced intestinal lipid absorption, improved glucose tolerance, enhanced insulin sensitivity, and reduced body weight. Mice fed a HFD were resistant against weight loss. Furthermore, sequencing of the gut microbiota revealed that inulin can enhance the diversity of the gut microbiota, reshaping the gut microenvironment, which in turn enables lipid absorption, ameliorates hepatic steatosis, and augments white fat. This study showed that inulin can improve metabolic syndrome in mice on a HFD, which can reduce intestinal lipid absorption associated with increased fecal bile acid excretion.

Globally, the number of obese people continues to grow. Studies predict that, by 2030, the number of obese people worldwide will reach 1.12 billion (Kelly et al., 2008; Ng et al., 2021). Furthermore, the prevalence rates of obesity-related metabolic syndromes, such as type 2 diabetes, steatosis, and hyperlipidemia, are increasing worldwide (Luukkonen et al., 2022). The problem of intestinal health, particularly among obese people, has attracted attention worldwide and is a hot topic (Correa et al., 2023). A high-fat Western diet absorbs large amounts of fat through the gut, which accumulates in organs such as the liver, white fat, brown fat, and the brain after absorption into the blood (Daskova et al., 2023). The aforementioned pathological process is involved in several metabolic diseases, such as hepatic steatosis, steatoinflammation, and insulin resistance (Huang et al., 2018; Liu et al., 2022). Recent evidence suggests that the gut microbiota plays a critical role in the control of obesity and NAFLD (Duranti et al., 2017; Soderborg et al., 2018). Microbial derivatives, including short-chain fatty acids, lipopolysaccharides, secondary bile acids, and indoles, have been shown to be involved in the regulation of liver lipid metabolism (Chen et al., 2023; Shi et al., 2023). The gut is extremely important for energy metabolism throughout the body, primarily maintaining a balance between absorption and excretion (Shin et al., 2022). Disruption of this balance leads to sub-optimal health, including disruption of the intestinal mucosal barrier caused by intestinal imbalance (Shin et al., 2022). Inulin, a plant-derived extract, is currently a hot topic in metabolic disease research. A recent clinical study of obese individuals showed that inulin can improve obesity-related metabolic disorders by improving the gut microbiota (Rodriguez et al., 2022).

Our study found that inulin reduced body weight, increased glucose tolerance, and improved insulin resistance in mice fed a regular diet. In addition, we showed that inulin reduced serum lipid levels in mice fed a normal diet, but no change in fat mass was observed. We believe that this evidence is sufficient to demonstrate the beneficial effects of inulin on this mechanism. Our findings are consistent with those of a previous study (Beisner et al., 2021), which showed that inulin improves the metabolic phenotypes by regulating the production of intestinal short-chain fatty acids. In addition, the protective effect of the intestinal mucosal barrier was enhanced by increasing the expression of intestinal antimicrobial peptides. We observed increased fecal bile acid content in inulin-fed mice fed a normal diet. Intestinal bile acids mediate intestinal absorption of lipids (Hu et al., 2023b). A recent study found that fecal bile acid excretion was positively associated with lower blood lipids (Wang et al., 2023). Bile acids simultaneously affect microbial survival and colonization by promoting lipid absorption (Wu et al., 2022). Therefore, we evaluated the metabolic phenotype inulin-fed mice fed a HFD to explore the underlying mechanisms. Compared with the control group, the inulin-fed HFD mice had the following phenotypes: decreased body weight, increased glucose tolerance, increased insulin sensitivity, decreased white fat content, and decreased liver lipid content. In addition, we observed an increase in fecal TG and bile acid content in inulin-fed mice fed a normal diet. These results suggested that inulin may reduce intestinal lipid absorption by promoting bile acid excretion in mice fed a normal diet. The increase in fecal TG excretion also supports this view. We further performed 16S sequencing analysis of feces from mice fed a normal diet and found that inulin increased the intestinal microbiota α-diversity and increased the ratio of *bacteroides*/firmicutes in mice. Previous studies have reported that the *bacteroides*/firmicutes ratio is a marker of gut microbial health, and a high proportion of *bacteroides*/firmicutes suggests a healthier gut microenvironment (Gu et al., 2017; Grigor’eva, 2020; Yang et al., 2020; Duan et al., 2021). However, we did not find changes in β-diversity, and we suggest that inulin only changed the composition of the gut microbes, not the species.

We found that inulin-fed mice on a HFD had lower serum total bile acid levels and higher fecal bile acid excretion. To this end, we examined the fecal TG content and found that the fecal TG content was significantly higher in inulin-fed mice fed a HFD (*p* < 0.05; Figure 4G). We further weighed the liver and fat of mice on a HFD and found that inulin reduced the weight of liver, inguinal fat (igWAT), and epididymal fat (EPWAT) (*p* < 0.05). The liver of the control group showed vacuolar degeneration and lipid accumulation in the cytoplasm, which showed typical hepatic steatosis. Inulin-fed mice on a HFD showed a complete reversal of these changes. We suggest that these changes may be due to decreased lipid accumulation in the liver as a result of a reduction in intestinal lipid absorption. We performed hematoxylin and eosin staining on the inguinal fat and found that inulin significantly reduced the diameter of the inguinal fat in mice fed a HFD. The inguinal fat size of inulin-fed mice on a HFD was found to be 40% smaller than that of the control group. Our findings are similar to recent reports (Du et al., 2020; Rodriguez et al., 2020; Zhu et al., 2020). The abovementioned evidence indicates a beneficial metabolic effect of inulin. We suggest that these results can be explained based on the decreased lipid absorption through increased bile acid excretion. The gut microbiota mediates the conversion of primary bile acids to secondary bile acid formation and mediates this process as a dominant factor (Guzior and Quinn, 2021). Therefore, we suggest that inulin causes increased bile acid excretion by remodeling the gut microbiota, which in turn affects the intestinal lipid absorption. To further determine the mechanism of bile acid reabsorption in the gut, we examined the expression level of FGF15, a gut bile acid reabsorption protein. We found that inulin significantly reduced the expression of the intestinal bile acid reabsorption protein FFG15 in mice fed a HFD. These results suggest that the inhibitory effect of inulin on the intestinal bile acid reabsorption protein FGF15 reduces the intestinal lipid absorption, which in turn affects lipid production and accumulation in the liver and white fat.

## 5 Conclusion

Inulin improves glycolipid metabolism by remodeling the gut microbiota and increasing fecal bile acid excretion in mice. In particular, inulin ameliorates the glycolipid metabolic phenotype of mice fed a normal diet, which includes reduced intestinal lipid absorption, improved glucose tolerance, enhanced insulin sensitivity, and reduced body weight. These changes may be caused by an increase in bile acid excretion resulting from changes in the gut microbiota that affect intestinal lipid absorption.

## Data Availability

The original contributions presented in the study are publicly available. The original sequencing data can be found in NCBI, the accession number is: PRJNA993596, https://www.ncbi.nlm.nih.gov/search/all/?term=PRJNA993596.
